# Treatment of Irradiated Mice with High-Dose Ascorbic Acid Reduced Lethality

**DOI:** 10.1371/journal.pone.0117020

**Published:** 2015-02-04

**Authors:** Tomohito Sato, Manabu Kinoshita, Tetsuo Yamamoto, Masataka Ito, Takafumi Nishida, Masaru Takeuchi, Daizoh Saitoh, Shuhji Seki, Yasuo Mukai

**Affiliations:** 1 Military Medicine Research Unit, Test and Evaluation Command, Ground Self-Defense Force, Setagaya, Tokyo, Japan; 2 Department of Immunology and Microbiology, National Defense Medical College, Tokorozawa, Saitama, Japan; 3 Department of Developmental Anatomy and Regenerative Biology, National Defense Medical College, Tokorozawa, Saitama, Japan; 4 Department of Ophthalmology, National Defense Medical College, Tokorozawa, Saitama, Japan; 5 Division of Traumatology, Research Institute, National Defense Medical College, Tokorozawa, Saitama, Japan; National Institutes of Health, UNITED STATES

## Abstract

Ascorbic acid is an effective antioxidant and free radical scavenger. Therefore, it is expected that ascorbic acid should act as a radioprotectant. We investigated the effects of post-radiation treatment with ascorbic acid on mouse survival. Mice received whole body irradiation (WBI) followed by intraperitoneal administration of ascorbic acid. Administration of 3 g/kg of ascorbic acid immediately after exposure significantly increased mouse survival after WBI at 7 to 8 Gy. However, administration of less than 3 g/kg of ascorbic acid was ineffective, and 4 or more g/kg was harmful to the mice. Post-exposure treatment with 3 g/kg of ascorbic acid reduced radiation-induced apoptosis in bone marrow cells and restored hematopoietic function. Treatment with ascorbic acid (3 g/kg) up to 24 h (1, 6, 12, or 24 h) after WBI at 7.5 Gy effectively improved mouse survival; however, treatments beyond 36 h were ineffective. Two treatments with ascorbic acid (1.5 g/kg × 2, immediately and 24 h after radiation, 3 g/kg in total) also improved mouse survival after WBI at 7.5 Gy, accompanied with suppression of radiation-induced free radical metabolites. In conclusion, administration of high-dose ascorbic acid might reduce radiation lethality in mice even after exposure.

## Introduction

After accidental exposure to high-dose radiation, casualties generally suffer from fatal damage to multiple organs, injuries that are termed acute radiation syndrome. Hematopoietic dysfunction is the most common complication of this syndrome, causing severe pancytopenia and fatal immune dysfunction. At present, short- or long-term therapy with cytokines such as granulocyte-colony stimulating factor (G-CSF), blood component transfusion, and even stem-cell transplantation depending on estimated exposure doses, has been recommended in medical management of hematopoietic syndrome/dysfunction [1–3]. However, it is difficult to use cytokine therapy when the casualties’ exposure dose is uncertain and clinicians can only estimate injury based on a grading system assessing the response of neurovascular, gastrointestinal, and cutaneous systems in the prodromal phase [1]. In addition, the optimal administration time and dose of cytokine therapy remains controversial. Swift administration might be restricted by adaptation criteria such as lymphocyte reduction after 48 h exposure in peripheral blood [4], although it is likely that administration should be initiated within approximately 24 h of exposure [1].

Medical countermeasures against radiation exposure have been classified into radioprotectants, radiation mitigators and radionuclide eliminators [2]. Amifostine (Ethyol, MedImmune Oncology, Inc., Gaithersburg, MD) is approved by the U.S. Food and Drug Administration [2]. These antioxidants are classified as radioprotectants [5]. Ascorbic acid is also a potent water soluble antioxidant in biological fluids [6] and might be used as a radio-protectant that effectively scavenges reactive oxygen species (ROS) formed during radiation exposure [7]. It has been reported that ascorbic acid effectively scavenges free radicals *in vitro* [7]. We previously demonstrated that pretreatment of mice with orally administered ascorbic acid prevented gastrointestinal syndrome following a lethal dose of radiation [8]. There have been many reports regarding the protective effects of antioxidants given prior to exposure; however, there are few studies of successful post-exposure treatment [9].

In the present study, we investigated the effects of high-dose ascorbic acid administered to mice after radiation exposure. We demonstrated that post-exposure administration of high-dose ascorbic acid (3 g/kg) reduced radiation-induced apoptosis in bone marrow cells and restored hematopoietic functions, thereby mitigating lethality in mice. In addition, post-exposure treatment with ascorbic acid was effective 24 h after irradiation. Divided post-exposure administration of ascorbic acid (1.5 g/kg × 2, immediately and 24 h after radiation, 3 g/kg in total) was also effective in treating the irradiated mice. Administration of high-dose ascorbic acid might be useful as a radioprotective therapy even after exposure.

## Materials and Methods

The Ethics Committee of Animal Care and Experimentation, National Defense Medical College, Japan, approved all requests for animal treatment and the intended procedures of the present study (Permission number: 12062). Treatment of all animals was in accordance with the Arrive Guidelines for Reporting Animal Research [10] (S1 ARRIVE Checklist). Regarding the mouse survival study, we have determined that the humane endpoint occurred 60 days after irradiation. We monitored the condition of the mice every day (at 13: 00) for 60 days after irradiation. If the mice appeared to be dying due to severe weight loss (approximately >25% loss of body weight) during the monitoring period, they were euthanized with isoflurane to minimize suffering. After completion of the monitoring period (day 60 post-irradiation), mice were humanely sacrificed by lethal isoflurane anesthesia.

### Mice and whole body irradiation (WBI)

Male C57BL/6 mice were pur-chased from Japan SLC (Shizuoka, Japan) and were used at an age of 8 weeks (20 ± 2 g). The mice were acclimated for 1 week before WBI. They were housed in a temperature-controlled, HEPA-filtered environment, and they were provided food and acidified water ad libitum. For WBI, mice were randomly divided into each group and were placed in a specially designed well-ventilated acrylic container and were exposed to 7 to 8 Gy of WBI, given at a dose rate of 0.45 Gy/min at 150 kV and 5 mA (HITACHI MBR-1505R2, Tokyo, Japan). The beam was filtered through a 2 mm alu-minum board. After radiation, mice were administered ascorbic acid, returned to the animal facility, and routinely maintained for a period of 60 days.

### Administration of ascorbic acid

Ascorbic acid (Wako, Osaka, Japan) was dissolved in physiological saline solution (Otsuka Pharmaceutical Co., Tokyo, Japan). Its pH was adjusted to 7.35 by adding sodium bicarbonate as described elsewhere [11]. The adjusted ascorbic acid solution (60 mg/mL) was administered intraperitoneally (i.p.) to the mice immediately after or before radiation, or at 1, 6, 12, 24, 36 or 48 h after radiation. Because the administered dose of ascorbic acid was 1 to 4.5 g/kg body weight, administration volumes were approximately 0.3 to 1.5 mL depending on mouse weight. Control mice received saline alone.

### Measurement of hematological parameters and plasma levels of ascorbic acid and cytokines

Mice were anesthetized with Isoflurane. Blood samples were obtained from the retro-bulbar plexus using heparinized anticoagulant tubes (Drummond Scientific Company, Broomall, PA). Hematological parameters were measured using a fully automatic blood cell counter (PCE-210, Erma Inc., Tokyo, Japan). To assess ascorbic acid levels in the plasma, we measured total ascorbic acid and dehydroascorbic acid using an enzyme linked immunosorbent assay (ELISA; Shima Laboratories, Tokyo, Japan) according to the manufacturer’s protocols and/or using high performance liquid chromatography (HPLC) at the SRL laboratory (Tokyo, Japan). Plasma cytokine levels (IL-1β, IL-6, TNF-α and IFN-γ) were measured using a multiplex bead analysis system (Bio-Plex Pro Suspension Array System; BioRad Laboratories, Tokyo, Japan) on a Luminex Bio-Plex 200 System (Bio-Rad Laboratories), according to the manufacturer’s instructions.

### Pathological examination of the bone marrow

The mice were euthanized with Isoflurane 14 days after WBI to obtain the femurs. The femurs were fixed in 20% formalin for 2 days and then immersed for 3 weeks for decalcification. Slides were prepared from the processed specimens, and stained with hematoxylin and eosin.

### Immunohistochemical staining of caspase-3 in bone marrow cells

Mice were euthanized 6 h after WBI to obtain the femurs. The femurs were immersed in phosphate-buffered 4% paraformaldehyde at 4°C overnight, then decalcified in 0.5 M ethylenediamine tetraacetic acid (pH 7.5) for 3 weeks.　Immunohistochemical staining of caspase-3 was performed on tissue sections of formalin-fixed, paraffin-embedded mouse bone marrow. After deparaffiniza-tion, antigenic retrieval was performed by immersing the sections in 0.01 mM sodium citrate (pH 6.0) and heating in an autoclave (121°C) for 5 min. Subsequently, samples were incubated with a peroxidase-blocking reagent (Dako, Glostrup, Denmark) for 10 min. After incu-bation with 2.5% normal horse serum in PBS for 30 min, the slides were incubated overnight with polyclonal rabbit anti-caspase-3 antibody (Asp175, Cell Signaling Technology, Inc. Danvers, MA) at a 1:250 dilution in PBS in a humidified chamber at 4°C. After washing, the slides were incubated with peroxidase-conjugated secondary antibody (ImmPRESS reagent; Vector Laboratories, Burlingame, CA) for 45 min at room temperature. Reactions were visualized with 3, 3’-diaminobenzidine (DAB), and the slides were counterstained with hematoxylin. As a negative control, the incubation step with primary antibody was omitted. After taking a picture of each specimen slide (× 200), the number of caspase-3-positive cells was counted in each image.

### Measurement of free radical metabolites and ferric-reducing ability in the plasma

The d-ROMs test (Diacron, Grosseto, Italy) was used for the measurement of free radical metabolites in plasma [12, 13]. It is a spectrophotometric method that assesses overall oxidative stress by measuring total hydroperoxide levels, given that hydroperoxides are intermediate oxidative products of lipids, peptides, and amino acids. Briefly, 0.02 mL plasma was diluted in 1 mL acetate-buffered solution. Hydroperoxide groups react with the transition metal ions liberated from the proteins in the acidic medium, and are converted to alkoxyl and peroxyl radicals by the Fenton reaction. These newly formed radicals, the quantities of which are directly proportional to those of the peroxides, were trapped chemically with 0.02 mL chromogen (N, N-diethyl-para-phenylendiamine), leading to the formation of the radical cation of this chromogen. The purple color resulting from this reaction over time was monitored in a spectrophotometer (Wismarll FRAS4, Tokyo, Japan) at 505 nm. The results of this method were expressed in conventional units (Carratelli units [UCarr]). The ferric-reducing ability in plasma was also measured spectrophotometrically using the biological antioxidant power (BAP) test (Diacron, Grosseto, Italy) [14]. In brief, a colored solution containing ferric ions is reduced to ferrous ions by reduction of the sample, and the antioxidant activity of the sample is proportional to the measured decrease in absorbance. The BAP assays were performed on the FRAS 4 analyzer according to the manufacturer’s protocols.

### Statistical analysis

Survival rates were compared by the Wilcoxon signed rank test. Data from more than 3 groups were analyzed by non-repeated measures ANOVA with Dunnett’s test for comparison with the control. Unpaired two-tailed Student’s t-test was used to analyze data between 2 groups. *P* < 0.05 was considered to be significant. All values in figures indicate means and standard deviations.

## Results

### Post-exposure treatment of irradiated mice with ascorbic acid promoted their survival

**Fig 1:**
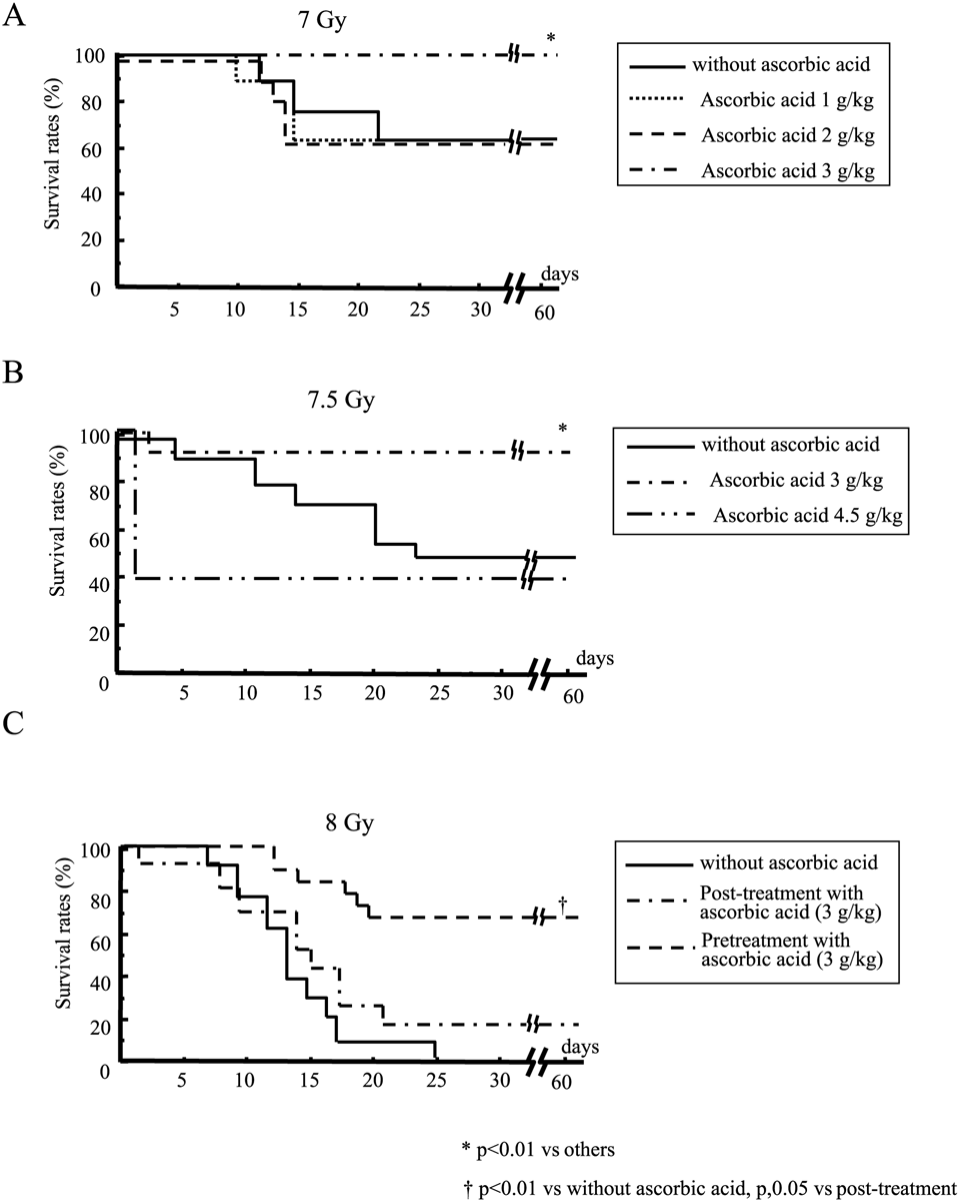
Survival of mice given post-exposure treatment with ascorbic acid after WBI at 7 Gy (A), 7.5 Gy (B), and 8 Gy (C). Mice were treated with the indicated doses of ascorbic acid immediately after radiation (A–C) or immediately before radiation (C). N = 15 in each group.

Mice died of severe weight loss due to irradiation. When mice were subjected to 7, 7.5 or 8 Gy WBI, we observed survivals of 67%, 47% and 0%, respectively, in untreated animals. In contrast, post-exposure treatment with 3 g/kg ascorbic acid immediately after irradiation significantly increased mouse survival after 7 Gy WBI (100% survival). Neither 1 g/kg nor 2 g/kg ascorbic acid was effective (Fig. 1A). This post-exposure treatment with 3 g/kg of ascorbic acid also significantly improved mouse survival after 7.5 Gy WBI (Fig. 1B). However, treatment with 4.5 g/kg ascorbic acid remarkably reduced mouse survival 1 day after 7.5 Gy of WBI (Fig. 1B). Massive administration of ascorbic acid might be harmful for these irradiated mice because more than half of the mice died within 1 day of administration of 4.5 g/kg ascorbic acid even in the absence of irradiation (data not shown). Post-exposure treatment with 3 g/kg of ascorbic acid also rescued 20% of the mice after lethal 8 Gy WBI (Fig. 1C). In contrast, pretreatment with ascorbic acid (3 g/kg) remarkably improved mouse survival after 8 Gy radiation (65% survival, Fig. 1C), indicating that pretreatment with ascorbic acid had a potent radioprotective effect. Post-exposure treatment with 4 g/kg of ascorbic acid as well as 4.5 g/kg markedly reduced mouse survival 1 day after 8 Gy WBI (13% and 0%, respectively), suggesting a harmful effect due to the extremely high dose of ascorbic acid.

### Hematological parameters in mice after WBI

**Fig 2:**
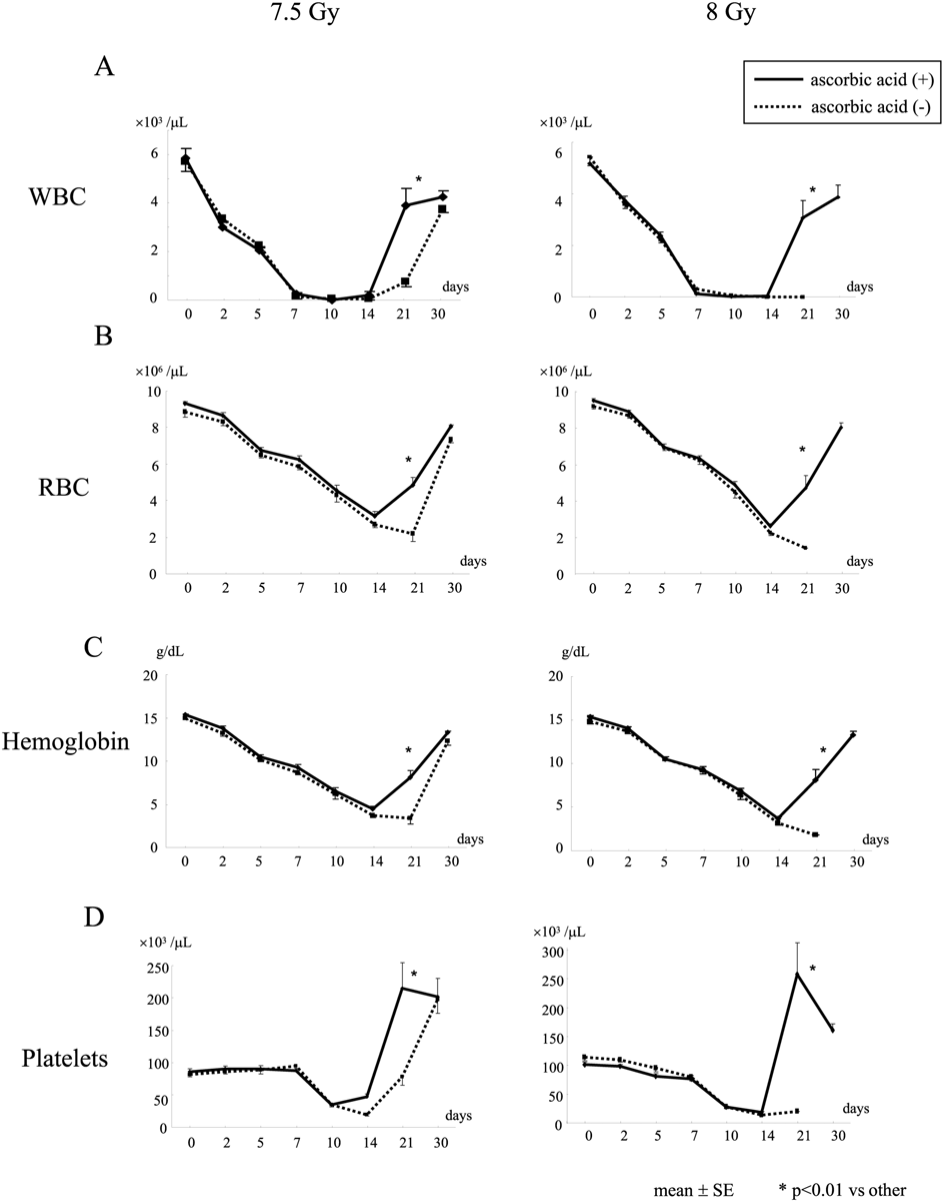
Changes in hematological parameters after irradiation of mice. Mice were treated with or without 3 g/kg of ascorbic acid immediately after WBI at 7.5 or 8 Gy. WBC (A), RBC (B), hemoglobin concentration (C), and platelets (D) were measured in mice at the indicated times. N = 10 in each group.

Post-exposure treatment with 3 g/kg of ascorbic acid did not restore white blood cells (WBC), red blood cells (RBC), hemoglobin levels, or platelet count in mice until 14 days after WBI at 7.5 or 8 Gy, while it markedly restored these hematological parameters 21 days after WBI (Fig. 2).

### Histological findings in the mouse bone marrow after WBI

**Fig 3:**
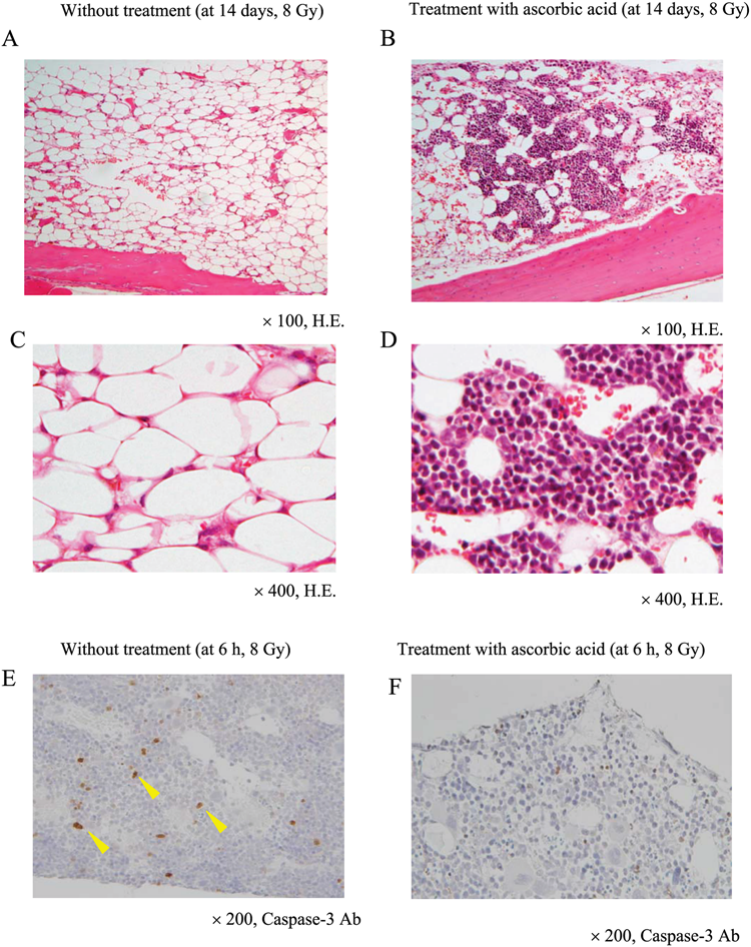
Histological findings in the bone marrow of irradiated mice. Mice were treated with or without 3 g/kg of ascorbic acid immediately after 8 Gy WBI. Bone marrows were obtained from the ascorbic acid-treated (A, C) or non-treated (B, D) mice 14 days after WBI and were stained with hematoxylin and eosin. Bone marrows were also obtained from the ascorbic acid-treated (E) or non-treated (F) mice 6 h after WBI to stain with anti-caspase-3 antibody. The images shown are representative of each group (n = 5).

We examined the bone marrow of mice 14 days after WBI. At that time, lethal 8 Gy radiation had severely damaged bone marrow cells (Fig. 3A, C). However, post-exposure treatment with 3 g/kg ascorbic acid preserved a portion of the bone marrow cells from the lethal radiation (Fig. 3B, D), although hematological parameters were still suppressed after 14 days (Fig. 2 right column). Immunohistochemistry showed that caspase-3-positive cells were increased in the bone marrow 6 h after WBI at 8 Gy (Fig. 3E, indicated by arrows). However, post-exposure treatment with 3 g/kg of ascorbic acid reduced the frequency of caspase-3-positive cells compared to that of the non-treated group (28 ± 3 vs 51 ± 6 cells/picture, p < 0.01) (Fig. 3F), suggesting that post-exposure treatment with ascorbic acid suppressed radiation-induced apoptotic cell death in the bone marrow. We also confirmed that 7.5 Gy WBI substantially damaged bone marrow cells, whereas post-exposure treatment with ascorbic acid (3 g/kg) significantly reduced this radiation-induced damage (data not shown).

### Plasma levels of ascorbic acid and BAP in mice after treatment with ascorbic acid

Administration of 3 g/kg ascorbic acid remarkably increased the plasma levels of ascorbic acid in mice with and without 7.5 Gy WBI 30 min and 1 h after administration (Table 1), and the levels were decreased after 2 h (irradiated mice, 120 ± 38; non-irradiated mice, 188 ± 27 μg/L at 2 h). Plasma BAP levels were also markedly increased 30 min after administration of ascorbic acid in both irradiated and non-irradiated mice (Table 1), suggesting that administration of ascorbic acid potently induced antioxidant capability in mice even after irradiation. Two hours after administration of ascorbic acid, increased plasma BAP levels decreased to normal levels in both irradiated and non-irradiated mice (Table 1).

**Table 1:** Plasma levels of ascorbic acid after radiation with or without ascorbic acid treatment.

**Radiation**	**Treatment**	**Plasma levels of ascorbic acid**		**Plasma levels of BAP**	
		**(μg/L)**		**(m Mol/L)**	
		**30 min after administration**	**1 h after administration**	**30 min after administration**	**2 h after administration**
7.5 Gy	Ascorbic acid (+)	3,659 ± 382*	1,878 ± 419*	30.1 ± 2.1*	5.7 ± 0.8*
	Ascorbic acid (-)	2.6 ± 0.7	1.7 ± 0.2	3.1 ± 0.1	3.4 ± 0.1
0 Gy	Ascorbic acid (+)	3,287 ± 520*	940 ± 128	28.3 ± 2.4*	5.3 ± 0.8
(no radiation)	Ascorbic acid (-)	1.2 ± 0.2		3.0 ± 0.1	

Mice were or were not treated with 7.5 Gy WBI. Subsequently, 3 g/kg ascorbic acid or saline was administered. Data show means ± SE from 5 mice.

*, p < 0.01 vs ascorbic acid (-).

### Plasma cytokine levels in mice after WBI

**Fig 4:**
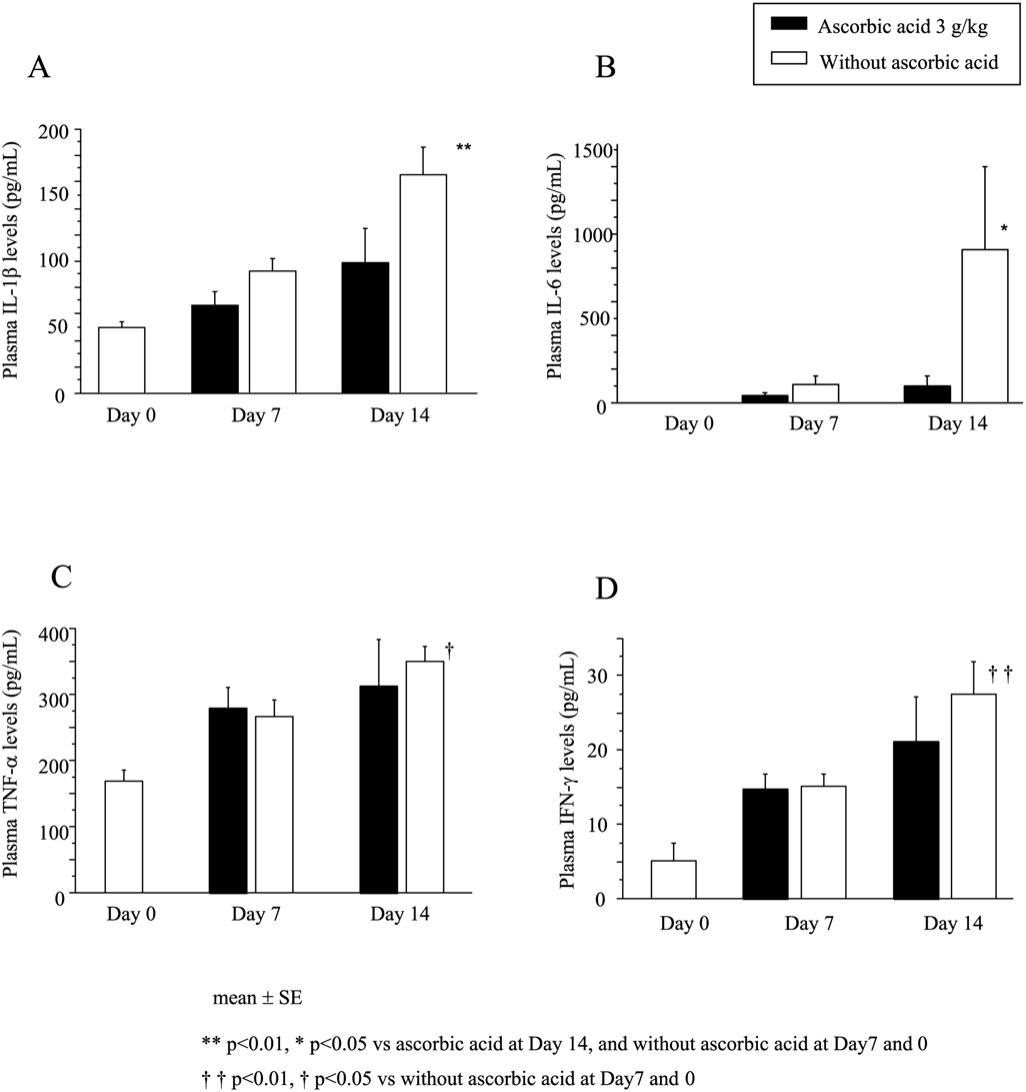
Change in plasma cytokine levels after radiation treatment of mice. Mice were treated with or without 3 g/kg ascorbic acid immediately after 7.5 Gy WBI. Plasma IL-1β (A), IL-6 (B), TNF-α (C) and IFN-γ (D) levels were measured at 0 (before radiation), 7 and 14 days after WBI. N = 5 in each group.

Plasma levels of IL-1β, IL-6, TNF-α and IFN-γ were significantly increased in mice 7 days after 7.5 Gy WBI and were further increased after 14 days (Fig. 4). However, post-exposure treatment with 3 g/kg of ascorbic acid significantly reduced the increase in plasma IL-1β and IL-6 levels after 14 days (Fig. 4A, B) and also tended to reduce the plasma IFN-γ levels but not TNF-α levels (Fig. 4D). Post-exposure treatment with ascorbic acid might mitigate the radiation-induced inflammatory cytokine response in mice.

### Period between irradiation of the mice to their treatment with ascorbic acid

**Fig 5:**
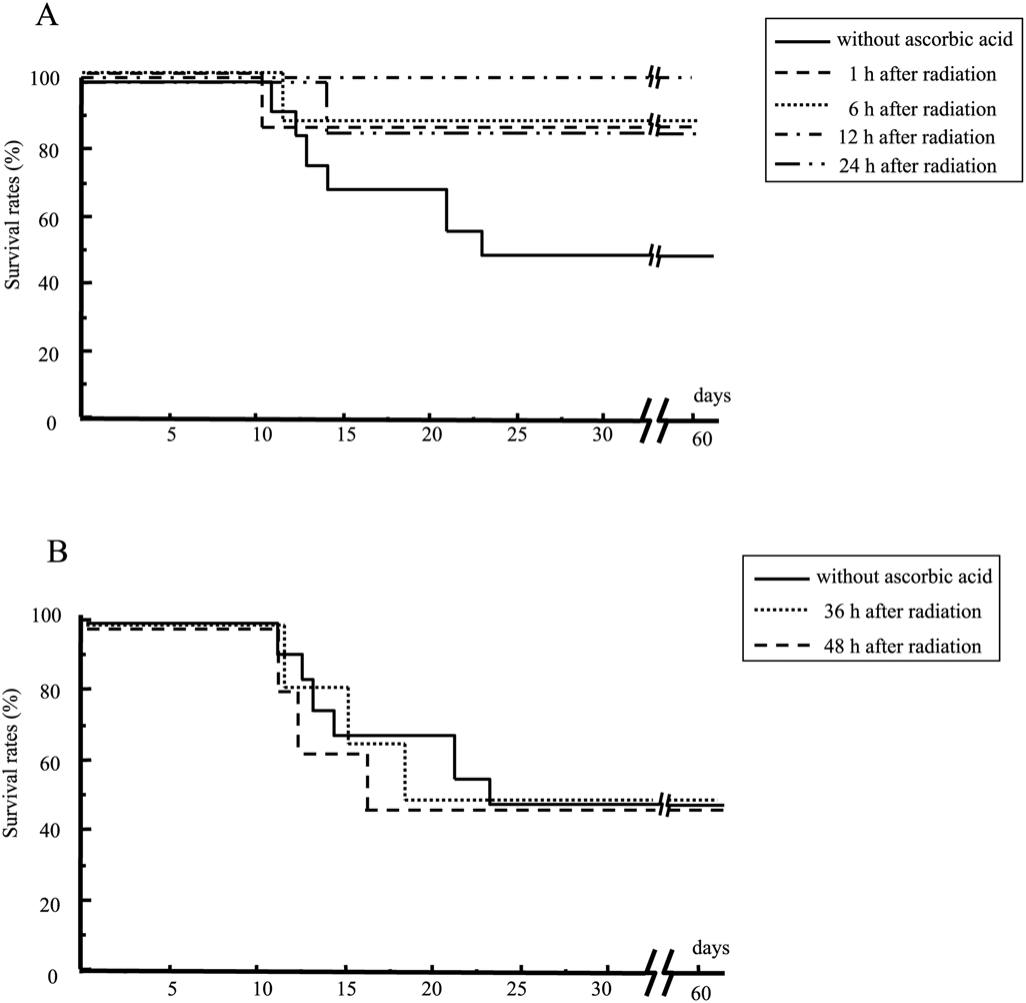
Mouse survival as a function of the time of post-exposure treatment with ascorbic acid. Mice were subjected to 7.5 Gy WBI. They were subsequently treated with 3 g/kg of ascorbic acid after 1, 6, 12 or 24 h (A) or 36 or 48 h (B). N = 15 in each group.

We examined how long we could delay post-exposure treatment with ascorbic acid after WBI. Ascorbic acid was administered to the mice at 1, 6, 12, 24, 36 or 48 h after 7.5 Gy WBI. Treatment with ascorbic acid up to 24 h after radiation effectively increased the survival of irradiated mice (Fig. 5A). However, treatment beyond 36 h post-irradiation was ineffective (Fig. 5B). The mice died of severe weight loss due to their exposure to radiation.

### Post-exposure treatment of irradiated mice with 2 doses of ascorbic acid

**Fig 6:**
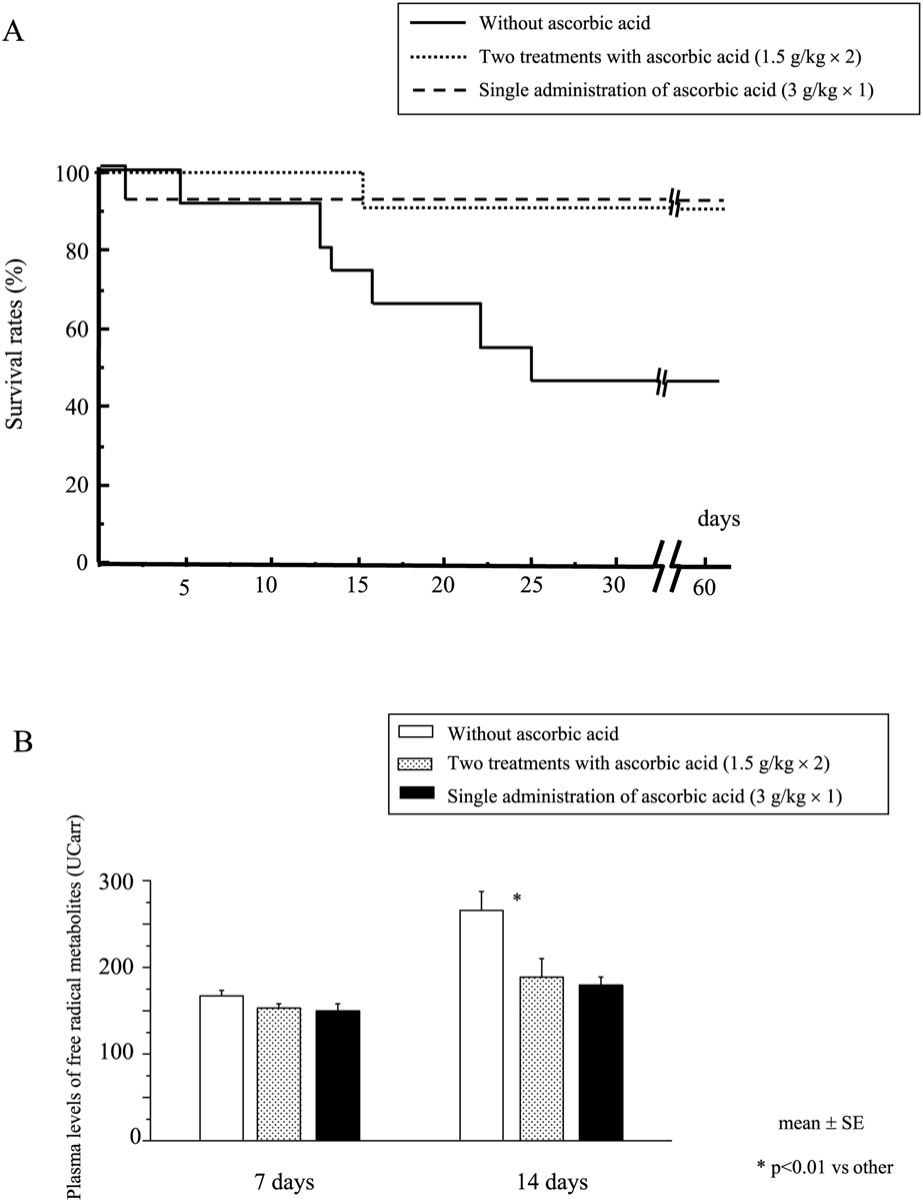
Survival of mice following 1 or 2 treatments with ascorbic acid (3 g/kg) after WBI. After receiving 7.5 Gy WBI, mice were treated with 2 courses of ascorbic acid (1.5 g/kg × 2, immediately and 24 h after radiation, 3 g/kg in total), or a single administration (3 g/kg, immediately after radiation), or without administration, and their survival was monitored (A). Plasma levels of free radical metabolites were also measured 7 and 14 days after WBI (B). N = 15 in each group.

When we consider ascorbic acid treatment in a clinical setting, a single administration of 3 g/kg of ascorbic acid appeared to be a high dose for mice to tolerate, although no non-irradiated mice died after this dose. We examined the efficacy of 2 treatments of ascorbic acid (1.5 g/kg × 2, immediately after radiation and 24 h after, 3 g/kg in total). Two treatments with ascorbic acid significantly increased mouse survival after WBI at 7.5 Gy (Fig. 6A), although neither single injection with 1.5 g/kg of ascorbic acid immediately after radiation nor 24 h after radiation was effective (data not shown). Furthermore, 2 treatments with ascorbic acid (1.5 g/kg × 2) as well as a single administration (3 g/kg) also significantly suppressed the elevation of plasma levels of free radical metabolites after radiation (Fig. 6B), suggesting an effective reduction of radiation-induced free radicals. All subject mice died of severe weight loss due to their exposure to radiation.

## Discussion

Although our previous studies clearly demonstrated the significant radioprotective effect of ascorbic acid on acute radiation syndrome (ARS), in particular gastrointestinal syndrome, pretreatment was indispensable to achieve its potent radioprotective effect [8, 15]. Here, we showed that pretreatment with ascorbic acid markedly reduced radiation lethality in mice subjected to lethal WBI at 8 Gy (Fig. 1C). However, it is impossible to pretreat ARS victims in most accidental cases. Therefore, we explored the possibility of using post-exposure treatment with ascorbic acid as a therapy for ARS. In our recent study, we demonstrated that adding post-exposure treatment with ascorbic acid to its pretreatment could achieve 100% survival in mice after lethal abdominal irradiation, although pretreatment alone supported only 20% survival [15]. This finding might imply possible beneficial effects of post-exposure treatment with ascorbic acid on irradiated subjects. Although the post-exposure treatment with ascorbic acid did not appear to be markedly effective in mice against lethal WBI at 8 Gy (20% survival) (Fig. 1C), it achieved significant effectiveness after milder WBI, i.e., 7 or 7.5 Gy (Fig. 1A, B).

Radiation-evoked radicals such as ROS have extremely short half-lives (nanoseconds). These radicals interact with various biological molecules, and some break DNA chains [16]. These short-lived radicals probably play crucial roles in modulating radiation-induced biological effects on tissues and cells, including apoptotic cell death [17]. In line with this, pretreatment with ascorbic acid can effectively scavenge ROS that are generated by radiation immediately after exposure, although its post-treatment cannot. On the other hand, although the exact underlying mechanisms responsible for radiation-induced injuries remain unclear, several reports have implicated an inflammation-based process in which cytokines and/or ROS are responsible [18–20]. Post-exposure inflammatory processes causing production of secondary ROS appears to be a plausible explanation why post-exposure therapy with ascorbic acid can be effective. In line with this, post-exposure treatment with ascorbic acid reduced the radiation-induced elevation of inflammatory cytokines (Fig. 4) and also elevation of free radical metabolites (Fig. 6B).

When subjects are exposed to a high dose of lethal radiation, transplantation of hematopoietic stem cells is recommended rather than treatment with high-dose ascorbic acid [21]. However, hematopoietic stem cell transplantation would be limited in the setting of a large scale nuclear disaster because large numbers of casualties would require treatment. In contrast, therapy with high-dose ascorbic acid would be possible if a large number of victims required treatment. Treatment with ascorbic acid could be effective and useful for mass casualties especially when radiation exposures were mild to moderate. In addition, post-exposure treatment with ascorbic acid can be effective even 24 h after radiation (Fig. 5). This application would be extremely helpful in the setting of a widespread nuclear disaster in which only limited medical help was available.

Administration of more than 4 g/kg ascorbic acid led to adverse effects in both the irradiated and non-irradiated mice. Because the pH of the ascorbic acid solution was adjusted with sodium bicarbonate, administration of a large amount of ascorbic acid might have caused sodium overload in mice. High-osmolarity ascorbic acid solution might act like other osmotic diuretics that induce anuria, dehydration, severe pulmonary congestion or pulmonary edema, and a fixed low cardiac output. Notably, 2 treatments with 1.5 g/kg of ascorbic acid immediately and 24 h after radiation achieved a potent radioprotective effect in mice (Fig. 6A, B). There is a possibility that high-dose administration of ascorbic acid could be divided into multiple treatments without losing its beneficial effect.

A recent study reported that high-dose intravenous administration of ascorbic acid is widely used by Complementary and Alternative Medicine practitioners to treat diverse conditions, such as infections, autoimmune diseases, and cancer. In that study, the high dose intravenous ascorbic acid (average 28 g; range, 1–200 g) appeared to be safe [22]. Nevertheless, when we administer gram doses of ascorbic acid to patients, we should be cautious about side effects. Nephropathy due to oxalate, one of the main metabolites of ascorbic acid, has been reported in patients with renal impairment after receiving massive administration of ascorbic acid [23–25]. Patients with glucose-6-phosphate dehydrogenase deficiency also reportedly developed intravascular hemolysis after receiving massive administration of ascorbic acid [26, 27]. However, a recent clinical study has shown that intravenous administration of 1.5 g/kg of ascorbic acid 3 times weekly is safe and free of toxicity in cancer patients, when patients with renal failure or glucose-6-deficiency were excluded [28]. Divided administration of high-dose ascorbic acid in addition to careful exclusion of patients with renal failure or glucose-6-phosphate dehydrogenase deficiency may be important when clinical usage of high-dose ascorbic acid treatment is considered.

Post-exposure therapy with high-dose ascorbic acid did not mitigate bone marrow suppression 1 to 2 weeks after radiation, although it significantly restored bone marrow function after 3 weeks (Fig. 2A). Post-exposure therapy with ascorbic acid reduced apoptosis in the irradiated bone marrow cells 6 h after radiation (Fig. 3 F). Nevertheless, bone marrow suppression persisted for 2 weeks. In a clinical setting, preventive measures against infection might be crucial for several weeks for patients treated with high-dose ascorbic acid.

A report demonstrated that supplementation of an antioxidant diet (_L_-selenomethionine, sodium ascorbate, N-acetyl cysteine, α-tocopherol succinate, and co-enzyme Q10) starting 24 h after exposure could reduce radiation lethality in mice [9]. In our previous study, we also administered ascorbic acid orally. However, after exposure to significant WBI, individuals might experience nausea, vomiting and diarrhea, rendering oral administration inappropriate in this affected period. We also performed administration of ascorbic acid intraperitoneally in the post-exposure phase. Perioral intake of radioprotective agents is recommended as the route of pretreatment, whereas a parenteral route is recommended as a route of post-exposure treatment for victims exposed to a massive dose of radiation.

Although there are a few reports regarding the efficacy of post-exposure treatment with amifostine (20–30 min after radiation in an *in vitro* study) [29], the American Society of Clinical Oncology guidelines for cytoprotective agents have recommended that amifostine must be slowly administered intravenously and is typically given 15 to 30 min before radiotherapy [30]. N-acetyl-cysteine is a multifaceted non-specific small molecule antioxidant. It has reportedly protected mice from radiation-induced gastrointestinal damage in post-exposure treatment 2 h after abdominal radiation [31]. AEOL 10150 is also a small molecule antioxidant analogous to the catalytic site of superoxide dismutase and reportedly protected radiation-induced proctitis by post-exposure treatment 1 h after irradiation of the rectum [32]. Although the optimal timing of administration of antioxidants as a radiation countermeasure has not been determined, the present finding that post-exposure treatment with ascorbic acid retained its efficacy for 24 h after radiation is quite attractive.

## Supporting Information

S1 ARRIVE Checklist (PDF)
